# A method for identifying discriminative isoform-specific peptides for clinical proteomics application

**DOI:** 10.1186/s12864-016-2907-8

**Published:** 2016-08-22

**Authors:** Fan Zhang, Jake Y. Chen

**Affiliations:** 1 Department of Molecular and Medical Genetics, University of North Texas Health Science Center, Fort Worth, TX 76107 USA; 2 Wenzhou Medical University 1st Affiliate Hospital, Zhejiang Province, China; 3 Institute of Biopharmaceutical Informatics and Technology, Wenzhou Medical University, Zhejiang Province, China; 4 School of Informatics and Computing, Indiana University, Indianapolis, IN 46202 USA; 5 Indiana Center for Systems Biology and Personalized Medicine, Indianapolis, IN 46202 USA

## Abstract

**Background:**

Clinical proteomics application aims at solving a specific clinical problem within the context of a clinical study. It has been growing rapidly in the field of biomarker discovery, especially in the area of cancer diagnostics. Until recently, protein isoform has not been viewed as a new class of early diagnostic biomarkers for clinical proteomics. A protein isoform is one of different forms of the same protein. Different forms of a protein may be produced from single-nucleotide polymorphisms (SNPs), alternative splicing, or post-translational modifications (PTMs). Previous studies have shown that protein isoforms play critical roles in tumorigenesis, disease diagnosis, and prognosis. Identifying and characterizing protein isoforms are essential to the study of molecular mechanisms and early detection of complex diseases such as breast cancer.

However, there are limitations with traditional methods such as EST sequencing, Microarray profiling (exon array, Exon-exon junction array), mRNA next-generation sequencing used for protein isoform determination: 1) not in the protein level, 2) no connectivity about connection of nonadjacent exons, 3) no SNPs and PTMs, and 4) low reproducibility. Moreover, there exist the computational challenges of clinical proteomics studies: 1) low sensitivity of instruments, 2) high data noise, and 3) high variability and low repeatability, although recent advances in clinical proteomics technology, LC-MS/MS proteomics, have been used to identify candidate molecular biomarkers in diverse range of samples, including cells, tissues, serum/plasma, and other types of body fluids.

**Results:**

Therefore, in the paper, we presented a peptidomics method for identifying cancer-related and isoform-specific peptide for clinical proteomics application from LC-MS/MS. First, we built a Peptidomic Database of Human Protein Isoforms, then created a peptidomics approach to perform large-scale screen of breast cancer-associated alternative splicing isoform markers in clinical proteomics, and lastly performed four kinds of validations: biological validation (explainable index), exon array, statistical validation of independent samples, and extensive pathway analysis.

**Conclusions:**

Our results showed that alternative splicing isoform makers can act as independent markers of breast cancer and that the method for identifying cancer-specific protein isoform biomarkers from clinical proteomics application is an effective one for increasing the number of identified alternative splicing isoform markers in clinical proteomics.

**Electronic supplementary material:**

The online version of this article (doi:10.1186/s12864-016-2907-8) contains supplementary material, which is available to authorized users.

## Background

Clinical proteomics is the application of proteomic techniques to the field of medicine with the aim of solving a specific clinical problem within the context of a clinical study. In the past year significant commitments from research institute and development of clinical proteomics has been witnessed. The application of clinical proteomic research is growing rapidly in the field of biomarker discovery, especially in the area of cancer diagnostics. Clinical proteomics holds the potential of taking a snapshot of the total protein complement of a cell, or body fluid, and identifying proteins as potential biomarkers for the differentiation of disease and health [1]. The study of clinical proteomic may provide us with opportunities in more effective strategies for early disease detection and monitoring, more effective therapies, and developing a better understanding of disease pathogenesis [2]. Such studies may aim at earlier or more accurate diagnosis, improvement of therapeutic strategies, and better evaluation of prognosis and/or prevention of the disease. Although clinical proteomics currently mainly focuses on diagnostics and biomarker discovery, it includes the identification of new therapeutic targets, drugs and vaccines for better therapeutic outcomes and successful disease prevention. In addition, success for a clinical proteomics requires the communication among clinicians, statisticians/bioinformaticians and biologists [3].

Until recently, researches have viewed protein isoform as any of several different forms of the same protein, not as a new class of early diagnostic biomarkers for clinical proteomics. Protein isoforms are an essential mechanism employed by human cells to enhance molecular functional diversity encoded by the genome. For *protein isoforms*, we refer to proteins derived from allellic polymorphisms, mRNA alternative splicing, or post-translational modifications (PTM). Allellic polymorphisms in protein-coding genes commonly take the form of single nucleotide polymorphisms (SNPs) of genes. Alternative splicing occurs in 40–60 % human genes and works by selecting specific exons and sometimes even intronic regions of the gene into mature mRNAs. Posttranslational modifications of proteins include all chemical modifications after protein translation, e.g., phosphorylation, glycosylation, and ubiquination. Approximately 8 % of these isoforms, including both SNPs and alternative splicing, are generated during the process of transcribing the coding genes into mRNA. More than 90 % of protein isoforms are created through PTMs after the mRNA is translated into a protein.

Traditional methods have been used for protein isoform determination such as EST sequencing [4], Microarray profiling [5] (exon array [6], Exon-exon junction array [7]), mRNA next-generation sequencing [8, 9]. However, there are several limitations with these traditional methods. First, they all identify isoforms in the transcript level, not in the protein level. Therefore, they cannot determinate isoform quantitatively in protein level, especially for measurement of low concentrations in biological specimens. Second, they give no connectivity information about the connection of nonadjacent exons. Third, they give no SNPs information about each exon and intron. Fourth, they give no information about posttranslational modifications of peptides. Last, the biggest challenge for the analysis of protein isoform with traditional methods is their low reproducibility by other methods such as RT-PCR. Only very few events are identified with high confidence. Indeed, the typical output is usually in the order of 10 validated alternative splicing events, which cannot meet the requirement of high through identification of protein isoform.

Recent advances in clinical proteomics technology, for example, LC-MS/MS, have enabled it possible to detect complex mixtures of proteins, peptides, carbohydrates, DNA, drugs, and many other biologically relevant molecules unique to disease processes [10] in parallel in biological samples. A modern mass spectrometry (MS) instrument consists of three essential modules: an ion source module that can transform molecules to be detected in a sample into ionized fragments, a mass analyzer module that can sort ions by their masses, charges, or shapes by applying electric and magnetic fields, and a detector module that can measure the intensity or abundance of each ion fragment separated earlier. Tandem mass spectrometry (MS/MS) has the additional analytical modules for bombarding peptide ions into fragment peptide ions by pipeline two MS modules together, therefore providing peptide sequencing potentials for selected peptide ions in real time. Recent developments of new generations of mass spectrometers and improvements in the field of chromatography have revolutionized protein analytics. Particularly the combination of liquid chromatography as a separation tool for proteins and peptides with tandem mass spectrometry as an identification tool referred to as LC-MS/MS has generated a powerful and broadly used technique in the field of proteomics [11]. LC-MS/MS proteomics have been used to identify candidate molecular biomarkers in diverse range of samples, including cells, tissues, serum/plasma, and other types of body fluids. For example, Flaubert et al. discovered highly secreted protein biomarkers which changed significantly in abundance corresponding with aggressiveness by using LC-MS/MS to analyze the secreted proteomes from a series of isogenic breast cancer cell lines varying in aggressiveness: non-tumorigenic MCF10A, premalignant/tumorigenic MCF10AT, tumorigenic/locally invasive MCF10 DCIS.com and tumorigenic/ metastatic MCF 10CA cl. D. They obtained proteomes from conditioned serum-free media, analyzed the tryptic peptide digests of the secreted proteins using a Waters capillary liquid chromatograph coupled to the nanoflow electrospray source of a Waters Q-TOF Ultima API-US mass spectrometer, and separated peptide on a C18 reverse phase column [12].

Although clinical proteomics can provide better evaluation of prognosis and prevention of the disease, there exist the computational challenges of clinical proteomics studies: 1) low sensitivity of instruments leads to many false negatives detection of molecules, especially when the molecules exists in low abundance and is unable to monitor specific molecules at will that can be associated with key phenotypes (typical in Genomics or functional genomics assays); 2) high data noise (false positives) introduced by limitation of accuracy of instruments causes false identification of peptides or assignment to proteins based on single peptide evidence brings uncertainty to the value of individual peptides; and 3) high variability and low repeatability of proteomics experiments exists even in high-abundance proteins (variability within individuals under different physiological conditions, worse across individuals), and the degree of variability differs for different proteins.

Therefore, in the paper, we presented a peptidomics method for identifying cancer-related and isoform-specific peptide for clinical proteomics application from LC-MS/MS which can provide hopes for improving both the sensitivity (many abundant proteins could generate alternative splicing isoforms in a cancer) and the specificity (particular types of protein isoforms may be uniquely regulated in a given condition) of candidate cancer biomarkers for clinical proteomics. First, we built a Peptidomic Database of Human Protein Isoforms, then created a peptidomics approach to perform large-scale screen of breast cancer-associated alternative splicing isoform markers in clinical proteomics, and last performed four kinds of validations: biological validation (explainable index), exon array, statistical validation of independent samples, and extensive pathway analysis. Our results showed that alternative splicing isoform makers can act as independent markers of breast cancer and that the method we presented is an effective one for increasing the number of identified alternative splicing isoform markers in clinical proteomics.

## Methods

### Reagents

Ammonium carbonate, ammonium bicarbonate, urea, formic acid, lysozyme, 2-Iodoethanol, and triethylphosphine were all purchased from Sigma-Aldrich (St. Louis, MO, USA). Acetonitrile and MS grade water were purchased from Honey Burdick & Jackson (Morristown, NJ, USA). Trypsin was purchased from Worthington Biochemical Corporation (Lakewood, NJ, USA). Seppro tip IgY-12 and reagent kit were purchased from GenWay Biotech (San Diego, CA, USA).

### Human plasma samples

Plasma protein profiles were collected by the Hoosier Oncology Group (HOG) (Indianapolis, IN, USA) in two batches, which we refer to as Study II and III (each contained 40 plasma samples from women with breast cancer and 40 plasma samples from healthy age-matched volunteer women as control). Most of patients involved in the two studies were diagnosed with a stage II or III or earlier breast cancer. Most patients had previously been treated with chemotherapy. All samples were collected with the same standard operating procedure and stored in a central repository in Indianapolis, IN, USA. The demography and clinical distribution of breast cancer stages/subtypes for Study II and III are comparable (Additional file 1: Table S1). In Study II, there are 9 metastasis and 30 non-metastasis, 30 INV and 10 DCIS, mean tumor size 1.56, 8GI, 11 GII and 15 GIII. In Study III, there are 1 metastasis and 20 non-metastasis, 23 INV and 8 DCIS, mean tumor size 1.93, 3GI, 9GII and 18GIII.

### Proteomics methods

Biomarker identification and characterization holds great promise for more precise diagnoses and for tailored therapies. The heterogeneity of human cancers and unmet medical needs in these diseases provides a compelling argument to focus biomarker development in cancer. Mass Spectrometry (MS)- based proteomics approaches have provided insight into biomarkers of cancer and other diseases with femtomole sensitivity and high analytical precision. We presented a four steps pipeline for the identification and validation of isoform-specific peptide biomarkers from breast cancer proteomics: Peptide Search Database Construction, Peptide Identification and Quantification, Statistical Identification of Isoform Markers, and Validation.

### Peptide search database construction

A comprehensive database of human peptides characteristic of all known and theoretic protein isoforms was developed in three steps: 1) obtaining gene structures of all protein-coding genes in the human genome, 2) compiling in silico isoform junction peptides, and 3) validating those peptides in current protein knowledgebase.

First, we downloaded all information about human genes in the Ensemble [13]. We retrieved gene information such as name, position, exon phase, exon/intron coordinates, and annotation. Exons which overlap with each other were classified into a group, and a serial number was assigned to each group according to its order in the sequence. For instance, the first group in the gene would be marked as group one, and the second as group two, etc. Introns can be obtained by the sequence between two exons.

Then, we generated in silico Isoform Junction Peptides (IJP), which contains two types of peptides: the peptides translated from all exons and the ones that are virtually translated from all possible exon/intron junction regions. Four types of exon/intron sequence joining types are considered when generating IJPs: intron-exon (I_E_TH, left intron retention junction), exon-intron (E_I_TH, right intron retention junction), knowledgebase validated exon-exon (E_E_KB, exon-exon junctions which can be found in Ensembl transcripts) and theoretical exon-exon (E_E_TH, exon-exon junctions which cannot be found in Ensembl transcripts).

For those exons with the phase information in Ensemble transcript, we directly used the phase to translate the sequence. For those exons without the phase information in Ensemble transcript, we designed an artificial translation method as follows. This sequence is used to generate three peptides, each of which has a different opening reading frame (ORF) and a maximal length of 140 amino acid residues (longer than the longest possible peptide fragments directly obtained from a MS/MS spectrum). The three ORFs are estimated in a validation procedure, where the ORF will be discarded if a stop codon is found in exon, knowledgebase validated exon-exon, or theoretical intron-exon, or if a stop codon is found in the first exon in theoretical exon-exon or theoretical exon-intron.

In the third and final step, we validated each IJP in the ensemble transcript database. Those Ensemble predicted transcripts have been mapped by Ensemble to full-length or near-full-length protein sequence already available in the public sequence databases [13]. We labeled the IJP as knowledge based (_KB) if it can be matched as a substring of any ensemble transcript of the same ensemble gene; otherwise, as theoretic (_TH).

### Peptide identification and quantification

Proteins were prepared and subjected to LC/MS/MS analysis. Samples were run on a Surveyor HPLC (ThermoFinnigan) with a C18 microbore column (Zorbax 300SBC18, 1 mm × 5 cm). All tryptic peptides (100 μL or 20 μg) were injected onto the column in random order. Peptides were eluted with a linear gradient from 5 to 45 % acetonitrile developed over 120 min at a flow rate of 50 μL/min, and the eluant was introduced into a ThermoFinnigan LTQ linear ion-trap mass spectrometer. The data were collected in the “triple-play” mode (MS scan, Zoom scan, and MS/MS scan).

We searched the OMSSA against the protein isoform database we created to identify peptide. Peptide quantification was carried out using the LC/MS-based label-free protein quantification software licensed from Eli Lilly and Company. Label-free peptide identification and peptide quantitative analysis services were performed by professionals at the Protein Analysis and Research Center/Proteomics Core of Indiana University School of Medicine, co-located at Monarch Life Sciences, Inc, Indianapolis. For a thorough review of the principle and method developed and used, refer to the review by Wang *et al* [14]. Briefly, once the raw files were acquired from the LTQ, all extracted ion chromatograms (XIC) were aligned by retention time. Each aligned peak should match parent ion, charge state, daughter ions (MS/MS data) and retention time (within a 1-min window). If any of these parameters were not matched, the peak was disqualified from the quantification analysis. After alignment, the area-under-the-curve (AUC) from individually aligned peak was measured, normalized, and compared for their relative abundance using methods described in [15]. All peak intensities were transformed to a log2 scale before quantile normalization. Peptides with intensity lower than preset quality threshold are marked as present; otherwise, as absent.

### Statistical identification of isoform markers

Statistical Significance was measured by a three-step method. First, we conducted a Chi-Square Goodness-of-Fit Test to calculate the p value (also called false discovery rate). Then we calculated the FDR adjusted p value. Last, we calculated the FDR q value using the Storey-Tibshirani method [16]. We chose a significance screening filters (*q* < 0.05) to select peptides of which we estimated significant differences in the health and breast cancer samples. The False Positive Rate (FPR) or expected proportion of false positive among the proteins with declared changes is FPR = qvalue × number of the proteins with declared changes.

### Validation

Four validation methods including biological, statistical, Exon Array and pathway validation methods were used to validate our results. Biological validation was carried out with Explainable Index. For gene, we define “Explainable Index” as

$$ \alpha =\frac{\#co{n}_C+1}{\# in{c}_C+1}\cdot \frac{\#co{n}_H+1}{\# in{c}_H+1}, $$

where #*con* is the number of consistent peptide markers and #*inc* is the number of inconsistent peptide markers, *C* be cancer marker set and *H* be health marker set. If *α* > 1, we define the gene to be “more explainable”; and if *α* ≤ 1, we define the gene to be “less-explainable”.

The “consistent” is defined as one of following three conditions:

1. ∃*i*, *j*, *k*:*E*_*j*_ ∊ *H&E*_*i*__*E*_*k*_ ∊ *C*(*i* < *j* < *k*)
2. ∃*j*:*E*_*j*_ ∊ *H* and ∄ *E*_*i*__*E*_*k*_(*i* < *j* < *k*)
3. ∃*i*, *k*:*E*_*i*__*E*_*k*_ ∊ *C*(*i* < *k*) and ∄ *E*_*j*_(*i* < *j* < *k*);

And the “inconsistent” is defined as one of following three conditions:

1. ∃*i*, *j*, *k*:*E*_*j*_ ∊ *C&E*_*i*__*E*_*k*_ ∊ *H*(*i* < *j* < *k*)
2. ∃*j*:*E*_*j*_ ∊ *C* and ∄ *E*_*i*__*E*_*k*_(*i* < *j* < *k*)
3. ∃*i*, *k*:*E*_*i*__*E*_*k*_ ∊ *H*(*i* < *k*) and ∄ *E*_*j*_(*i* < *j* < *k*).

For statistical validation, we used forward feedback neural network to train Study II and then test Study III. We chose each combination of N (*N* = 5 for five-marker panel or *N* = 10 for ten-marker panel or *N* = 26 for twenty-six-marker panel) out of all the 26 differentially expressed isoforms common in both Study II (90) and B (79) as inputs to the FFNN. The training sets are 40 healthy and 40 cancer samples from Study II. The testing sets are independent 40 healthy and 40 cancer samples from Study III.

For a neural network, the output data has to be transformed into binary or numerical data. A two-variable outcome encoding scheme, i.e., healthy = (0,1), cancer = (1,0) was used. In this scheme, it is theoretically possible to have (1,1) or (0,0) as outcomes although extremely rare. For the two variable outcome encoding scheme, we constructed the input layer as N nodes (corresponding to a N-marker panel outcome), the hidden layer as 7 nodes, and the output layer as 2 nodes.

In order to find the optimal classifier, we presented an optimization method that measures the area under the curve (AUC) for Receiver Operating Characteristics (ROC). In this scheme, we first trained neural network for each combination using Study II results. Then, we measured the AUC for each combination using Study III results for testing. Lastly, the optimal combination C^*^ was determined by

$$ {C}^{*}=\underset{c}{ \arg \min }AUC\left(NE{T}_C,P\right), $$

where AUC is the area under the ROC curve of neural network’s prediction result, NET is the trained neural network, C is combination of picking N out of the 26 isoforms, and P is the testing set of Study III.

The Exon Array for validation was downloaded from GSE19154 in Gene Expression Omnibus. R and BioConductor libraries were used to perform Exon Array analysis. For pathway validation, the 90 alternative splicing biomarkers in Study II and the 79 alternative splicing biomarkers in Study III were used to perform pathway analysis using the Kyoto Encyclopedia of Genes and Genomes (http://www.genome.ad.jp/kegg/) [17]. Significance level for pathway comparisons was set by hit number >2 due to results of small counts. This allows avoiding any assumptions about the shape of sampling distribution of population.

## Results

The statistics of IJP are shown in Table 1. Among the 5060822 peptides we derived, there are 208269 exon sequences, 222731 validated exon-exon junctions, 4109197 hypothetical exon-exon junctions, 413761 exon-intron junctions and 106864 intron-exon junctions. There are 367956 normal exon-exon junctions, in which the combined exons are continuous on the gene sequence, and 3963972 skipping exon-exon junctions. The longest exon peptide length is 6057 amino acids (aa), and the average exon length is 48 aa; the longest junction peptide length is 140 aa, and the average junction peptide length is 64 aa.

**Table 1:** The statistics of peptide database

Peptide Type	Number of Peptides	E_E Type	Number of Peptides
EXON_KB	208269	Normal E_E	367956
E_E_KB	222731	Skipping E_E	3963972
E_E_TH	4109197	Peptide Length (aa)	
E_I_TH	413761	Longest Exon	6057
I_E_TH	106864	Average Exon	48
		Longest Junction	140
Total	5060822	Average Junction	64

Among the total 5060822 peptides, intron-exon junctions account for the largest proportion, and theoretical exon-exon junctions the smallest proportion. Majority of exon-exon junctions are normal, while the minority are exon skipping. The average lengths are 64 and 48, for junction and exon, respectively. The maximum of length are 140 and 6057, for junction and exon, respectively. The peptide types are exon region (EXON_KB), annotated exon-exon junctions (E_E_KB), hypothetical exon-exon junctions (E_E_TH), hypothetical exon-intron junctions (E_I_TH), and hypothetical intron-exon junctions (I_E_TH)

### Alternative splicing peptides searching

In order to identify the tumor-specific alternative splicing isoform patterns, we ran OMSSA search engine with the peptide database against 40 normal plasma and 40 breast cancer plasma in Study II. Maximum 1 missed cleavage, Maximum 10 peptide hitlist length per spectrum, and Evalue cutoff 1.0 were chosen for filtering peptides.

### Statistics analysis

**Fig. 1:**
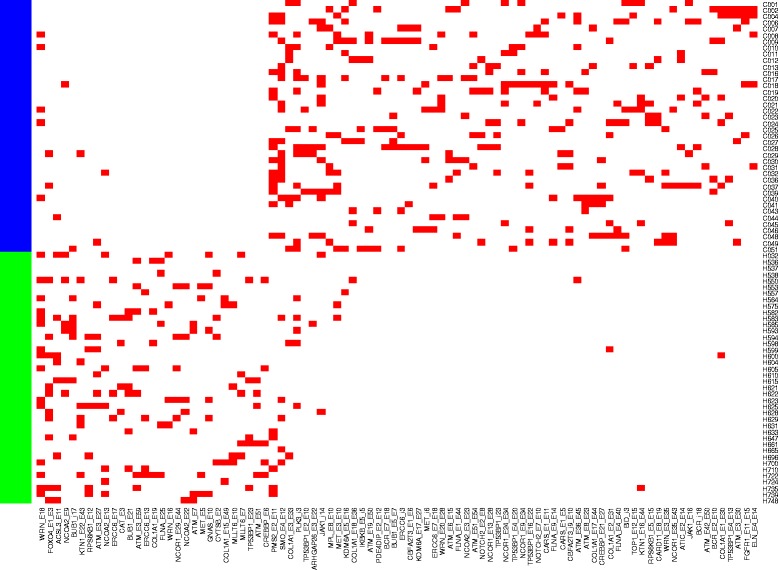
Heatmap of 90 alternative splicing isoform markers differentiating the normal and cancer samples of Study II. X axis is 90 alternative splicing isoform markers. Y-axis shows the cancer and normal samples ordered by unsupervised clustering. The top are cancer samples and bottom normal samples (H, health, green; C, cancer, blue). Red squares stand for presence, and white ones for absence

**Fig. 2:**
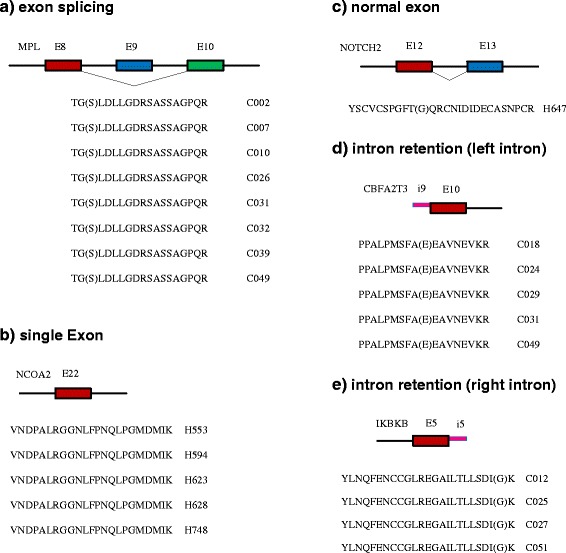
Five splicing types. Red, blue and green boxes are exon. Pink boxes are retained intron. Black lines are intron

**Fig. 3:**
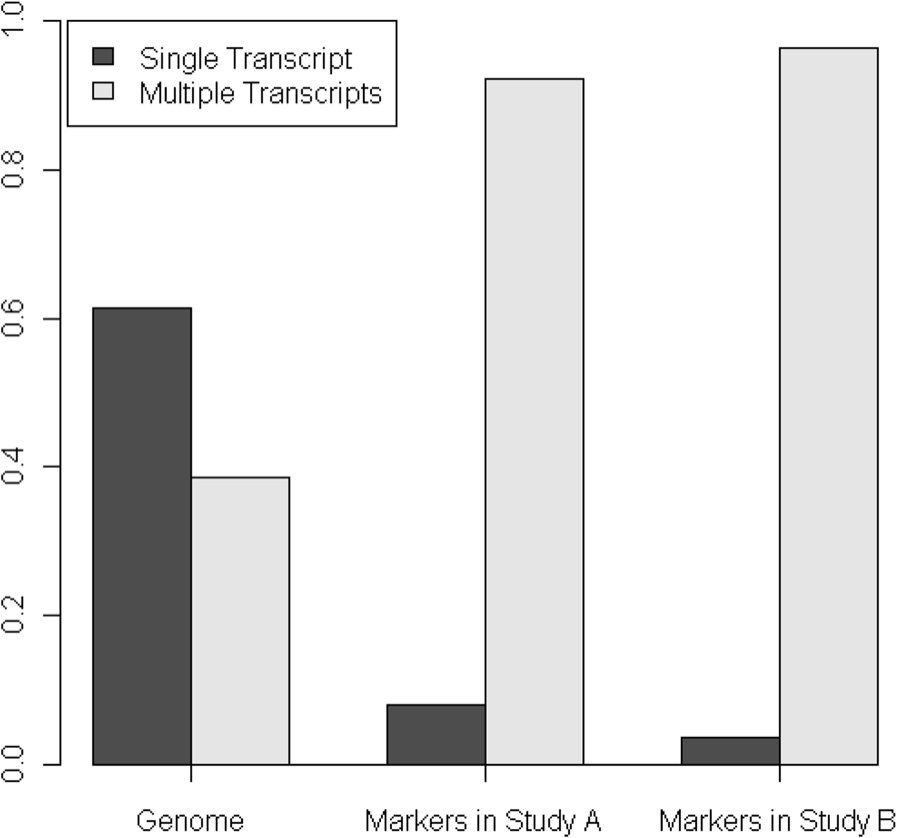
Densities for genes with single transcript and multiple transcripts across whole genome, Study II’s markers and Study III’s markers. It shows that alternative splicing isoform markers could be more likely to be found for genes with two or more than two transcript variants encoding different isoforms than genes with only one transcript (Chisquare Pvalue =1.35e-11 between genome and Study II’s markers, Chisquare Pvalue =0 between genome and Study III’s markers)

With the statistics analysis in the method section, 90 alternative splicing isoforms in 38 genes were found, which showed statistically significant (*q* < 0.05) differences between normal breast and breast cancer samples in Study II (Additional file 2: Table S2; Fig. 1). Four out of five kinds of alternative splicing isoforms: exon splicing, single Exon, intron retention (left intron), and intron retention (right intron) were identified (Fig. 2) except for the normal exon for which we fail to reject the null hypothesis that there is no difference between normal and cancer samples since the p-value is not less than the significance level. Among the 90 alternative splicing isoforms, 57 are exon splicing, 23 single Exon and 10 intron retention. Those exon splicing and intron retention markers are more likely to be present in cancer samples than in normal samples and those single exon markers are more likely to be present in normal samples (*χ*^2^ = 53, df = 1, pvalue = 3.2e-13, Table 2). Another interesting finding is that Alternative Splicing isoform markers could be more likely to be found for genes with two or more than two transcript variants encoding different isoforms than genes with only one transcript (Chisquare Pvalue =1.35e-11 between genome and Study II’s markers, Chisquare Pvalue =0 between genome and Study III’s markers, Fig. 3). The human genome contains totally 30370 genes with only one transcript and 19136 genes with two or more than two transcript variants. Isoform Markers in Study II contains totally 3 genes with only one transcript and 35 genes with two or more than two transcript variants. Isoform Markers in Study III contains totally 2 genes with only one transcript and 53 genes with two or more than two transcript variants.

**Table 2:** number of alternative splicing and normal markers between the normal and cancer samples

	Health	cancer	Total
Alternative Splicing	7	60	67
Normal	22	1	23
total	29	61

### Four validation methods

We presented four validation methods to validate our results. First we used the explainable index defined in method section to perform biological validation for the 38 gene markers. 36 out of 38 genes are “more explainable” except for two genes:JAK1 and KTN1 with explainable index of 1. The mean explainable index is 3.526316, the median explainable index 2, and maximum 12.

We then performed the validation using the Human Exon 1.0 ST Array we downloaded from GSE19154 in Gene Expression Omnibus. The experiments include six mRNA samples which were extracted from human breast cancer cell line MCF7, and MCF10A, a nontumorigenic human breast epithelial cell line.

Array analysis was performed using R and BioConductor libraries. Probeset in the exon array to the peptide sequence in our database was performed using the exon’s starting and ending positions in each transcript. Because of the limitation of the exon array, we can only validate the 23 single exon markers and test if those markers are more likely to be expressed in the same group as in our proteomics result. The validation results show that 21 of 23 single exon markers were confirmed by the exon array (Additional file 2: Table S2). The two unconfirmed markers were identified with not very significant pvalue (LLPNQNLPLDITLQSPTGAGPFPPIR 0.166; AAMKPGWEDLVRR 0.0895). The mutations that alter a splice site or a nearby regulatory sequence may have subtle effects by shifting the ratio of the resulting proteins without entirely eliminating any form, as a result of alternate splicing.

**Fig. 4:**
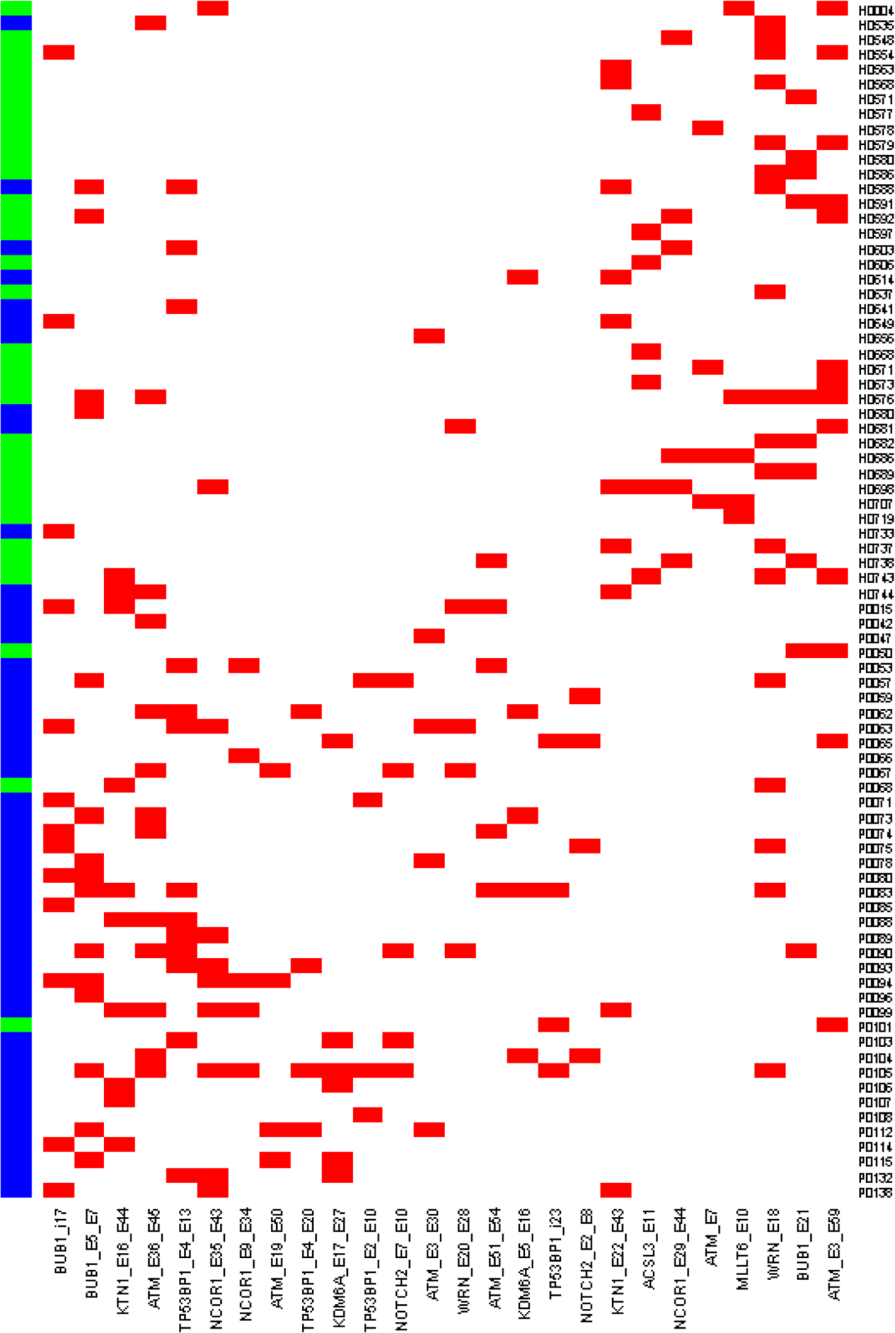
Heatmap of 26 alternative splicing isoform markers in Study II differentiating the normal and cancer samples of Study III. X axis is 26 alternative splicing isoform markers from Study II. Y-axis shows the cancer and normal samples in Study III ordered by unsupervised clustering. The top are health samples and bottom cancer samples. The prediction results are green for health and blue for cancer. Red squares stand for presence, and white ones for absence

Next, we performed the statistical validation using the independent 40 healthy and 40 cancer samples from Study III as testing set (Fig. 4). 66 (82.5 %) out of 80 samples are correctly predicted. For the 40 cancer samples, the prediction accuracy is 37/40 = 92.5 %.

### Pathway analysis

Last, we performed extensive pathway analysis to discover highly significant pathways from a set of cancer vs healthy samples. The knowledge of activation of these processes may lead to novel assays identifying their proteomic signatures in plasma of patient at high risk for cancer disease. In Study II, of the 24 significant pathways we observed, at least 23 of these pathways were involved cancers, signal transduction, diseases, and cellular processes (Additional file 3: Table S3). The top pathways include Pathways in cancer (8), MAPK signaling pathway (3), Cell cycle (3), Apoptosis (3), Focal adhesion (3), Adherens junction (3), Jak-STAT signaling pathway (3), Prostate cancer (3). All are also significant pathways in Study III except for Adherens junction (Additional file 4: Table S4). And ‘pathways in cancer’ are listed top 1 in both Study II and Study III.

## Discussion

In this study we developed a peptidomics approach to identifying novel protein isoforms for clinical proteomics application. First, we built a Peptidomic Database of Human Protein Isoforms, then created a peptidomics approach to perform large-scale screen of breast cancer-associated alternative splicing isoform markers in clinical proteomics, and last performed four kinds of validations: biological validation (explainable index), exon array, statistical validation of independent samples, and extensive pathway analysis. Our results showed that alternative splicing isoform makers can act as independent markers of breast cancer and that the method we presented is an effective one for increasing the number of identified alternative splicing isoform markers in clinical proteomics.

The combination of protein isoform database, statistical analysis, and statistical and biological validations has the potential for extremely high-resolution signatures to better resolve tumor subtypes and determining optimal therapies.

### Protein isoform database

With the advances in mass spectrometry (MS) and large-scale generation of MS/MS (tandem MS)-based proteomics data, it has become clear that MS-based peptide sequence data can be mined to identify and validate isoforms in the protein level rather than in the transcript level where traditional methods such as EST sequencing [4], exon array [6], Exon-exon junction array [7]), and mRNA next-generation sequencing [8, 9] do. Moreover, it can eliminate limitations with these traditional methods for protein isoform determination such as no connectivity about connection of nonadjacent exons, no SNPs and PTMs, and low reproducibility.

However, there are some limitations in identifying protein isoforms using current MS proteomics search database. For example, traditional mass spectrometry search database using isoforms of well-known proteins is biased. Using ESTs and a sequence database compression strategy to identify peptide isoforms existing in the EST database from MS data [18] is also defective because of the inherent characteristics of ESTs, such as transcript redundancy, low sequence quality and high error rates. 282 novel open reading frames were identified by searching six-frame translation of the human genome against MS spectrum [19]. But it only takes into account a small portion of alternative splicing isoform.

Although there are several general-purpose alternative splicing mRNA transcript databases including ASTD [20], EID [21, 22], ASPicDB [23], and ECgene [24], they cannot be used for searching uncharacterized protein isoforms. And also the coverage of splicing junctions in all the databases are small. The new PEPPI database [25] contains the five types of combinations of exon and intron: EXON_KB, E_E_KB, E_E_TH, E_I_TH and I_E_TH, and makes it easy for different types of biomedical users to search for and identify alternative splicing isoform from proteomics experiment. We believe that it will be useful in the ongoing analysis of proteomics data, particularly those with clinical application potentials. The current PEPPI database contains only alternative splicing isoform. We will add SNP protein isoform and PTM protein isoform in the future so that the database of virtual peptides will be expanded to accommodate the amino acid alterations introduced by each SNP and PTM.

### Biological significance of isoform-specific peptides

In this study, we have shown that isoform-specific peptides can distinguish normal breast from breast cancer. The number and type of splicing peptides identified exceeds the average number of events that is normally identified by splicing microarray profiling [26]. The accuracy and applicability of the newly identified alternative splicing signature was shown by its capacity to identify breast cancer sample (Fig. 4). The signature identified 92.5 % cancer samples and 72.5 % of normal samples in an independent set of 40 normal samples and 40 breast cancer samples. All cancer samples that were identified as normal could be either of the complexity of the proteome in plasma samples where the low abundance expected for specific markers of cancer are hindered, or of false positive associations that occur with analysis of high dimensional database.

We observed that there appeared to be a higher proportion of alternative splicing markers in cancer samples (58 out of 65 alternative splicing are predominant in the cancer samples) and a higher proportion of normal markers in normal samples (22 out of 23 normal splicing are predominant in normal samples). Those exon splicing and intron retention markers are more likely to be present in cancer samples than in normal samples and those single exon markers are more likely to be present in normal samples. The strong correlation of alternative splicing isoform with cancer suggests the potential value of alternative splicing as prospective markers for the early detection and treatment of cancer.

Interestingly, we also found that alternative splicing isoform markers could be more likely to be found for genes with two or more than two transcript variants encoding different isoforms than genes with only one transcript (Fig. 3).

Previously, many alternative splicing variants had been observed in cancer, for examples, EGFR, CD44, NER and BRCA1. In our 38 gene markers, alternative splicing events of at least 5 genes were previously reported to occur in cancer.

Two single exon markers and nine alternative splicing markers for ATM were identified in our results. This gene and the closely related kinase ATR are master controllers of cell cycle checkpoint signaling pathways that are required for cell response to DNA damage and for genome stability. Three alterations, del exon 4, deletion exon 29–34 and insertion of 137 bp in exon 46/47 were commonly observed in 8 HL cell lines and 7 clinical cases [27]. Katzenberger etc. presented the evidence that the ATM/CHK2 and ATR/CHK1 signaling pathways control gene expression by regulating alternative splicing [28]. Ho et. al. used ATM sequence alterations located within exons or in short intron regions flanking each exon that encompass putative splice site regions as predictor for late normal tissue responses in breast cancer patients treated with radiotherapy [29]. ATM allelic variants were reported to be associated to hereditary breast cancer in 94 Chilean women [30]. ATM SNPs have been associated with increased risk of breast, prostate, leukaemia, colon and lung cancer. Nguyen etc used two exons of ATM, both containing an SNP interfering with standard mutation scanning to screen 1356 subjects from an international breast cancer genetics Study IInd improved identification of rare known and unknown variants, while dramatically reducing the sequencing effort [31].

Three splicing markers (E3_E10 exon splicing, i6 intron retention and E5 single exon) for MET were identified in our results. The first two were predominant in cancer samples and the last one was predominant in health samples. Lee etc. had detected a novel type of structural variant of the tyrosine kinase receptor for MET, also known as the hepatocyte growth factor receptor, in mouse tissues, and demonstrated that a tyrosine kinase receptor could achieve additional diversity by alternative splicing at a key regulatory site in its cytoplasmic domain [32]. The cDNA of the variant transcript of MET lacks 141 base pairs and causes an in-frame deletion of 47 amino acids in the juxtamembrane region of the cytoplasmic domain. Extensive evidences indicate that MET signaling is involved in the progression and spread of several cancers such as breast, liver, lung, ovary, kidney, and thyroid [33]. And understanding of its role in disease has led to the development of Met as a major target in cancer drug and the development of a variety of MET pathway antagonists with potential clinical applications. Various mutations in the MET gene were reported to be associated with cancers. Zohar Tiran etc. identified a novel splice variant of the Met receptor, which encodes a truncated soluble form of the receptor [34]. This variant was produced as a recombinant Fc-fused protein named Cgen-241A and significantly inhibited HGF/SF-induced MET phosphorylation as well as cell proliferation, survival, and a profound inhibitory effect on cell scattering, invasion, and urokinase up-regulation.

CREBBP is ubiquitously expressed and is involved in the transcriptional coactivation of many different transcription factors. First isolated as a nuclear protein that binds to cAMP-response element binding protein (CREB), this gene is now known to play critical roles in embryonic development, growth control, and homeostasis by coupling chromatin remodeling to transcription factor recognition. Its alternative splicing results in multiple transcript variants encoding different isoforms. It was reported that Co-regulator expression of CREBBP/p300 had been associated with lower tumor grade [35]. We identified intron 14 of JAK1 was retained through translation, which might be related to the mutation of 14 exons of JAK1 [36]. Xie found 12 cases (14 %) found to have single nucleotide polymorphism in exon 14. Somatic mutations in the SMO gene have also been identified in breast cancer. Recently, two groups have shown that hedgehog signaling may be active in a subset of human breast cancer cell lines, and that SMO antagonists can inhibit breast cancer growth [37, 38].

## Conclusions

We developed a peptidomics method to discover novel alternative splicing biomarkers from breast cancer proteome. First, we built a Peptidomic Database of Human Protein Isoforms, then created a peptidomics approach to perform large-scale screen of breast cancer-associated alternative splicing isoform markers in clinical proteomics, and last performed four kinds of validations: biological validation (explainable index), exon array, statistical validation of independent samples, and extensive pathway analysis. Our results showed that alternative splicing isoform makers can act as independent markers of breast cancer and that the method we presented is an effective one for increasing the number of identified alternative splicing isoform markers in clinical proteomics.

## Additional files

Additional file 1: Table S1. Breast cancer plasma sources. (PDF 172 kb) Additional file 2: Table S2. 90 alternative splicing isoforms with statistically significant (*q* < 0.05) differences between normal breast and breast cancer samples in Study II (PDF 184 kb) Additional file 3: Table S3. Pathway analysis for Study II (XLS 34 kb) Additional file 4: Table S4. Pathway analysis for Study III (XLS 34 kb)
