# Supplementary material for: A method for identifying discriminative isoform-specific peptides for clinical proteomics application
Source: BMC Genomics. 2016 Aug 22;17(Suppl 7):522. doi: 10.1186/s12864-016-2907-8 (PMC5001247; doi:10.1186/s12864-016-2907-8)
Supplement: Additional file 1: Table S1. — Breast cancer plasma sources. (PDF 172 kb) [file 12864_2016_2907_MOESM1_ESM.pdf]

	Study A		Study B	
Age Distribution	30-39	>39	30-39	>39
# of patients	3	37	5	35
Race	3 – White<br>0- Black	36 – White<br>1– Black	3 – White<br>1- Black<br>1-others	32 – White<br>3– Black
Ethnicity	3 – Non-hispanic	36 – Non-hispanic<br>1 Hispanic	5 – Non-hispanic	34 – Non-hispanic<br>0 Hispanic<br>1- unknown
Metastasis	3 – No	27 – No<br>9 – Yes<br>1 – Unknown	3 – No<br>1-Yes<br>1-unknown	17 – No<br>10 – Yes<br>8– Unknown
Cancer Type*	2 – INV<br>1 – DCIS	28 – INV<br>9 – DCIS	1 – INV<br>3 – DCIS<br>1-unknown	22 – INV<br>5 – DCIS<br>8-Unknown
Tumor Size**	m = 1.15<br>[0.2,1.7]	m = 1.98<br>[0,5]	m = 1.3<br>[0.5,3.0]	m = 2.57<br>[0,5.4]
Tumor Grade***	1-GII<br>2-GIII	8-GI<br>10-GII<br>15-GIII<br>4-unknown	1-GII<br>3-GIII<br>1-unknown	3-GI<br>8-GII<br>15-GIII<br>9-unknown

* - INV: invasive; DCIS: ductal carcinoma in situ
** - the mean (m) is based on all defined values, including 0; the range shown excludes tumor size of 0
*** GI: tumor grade I, GII: tumor grade II, GIII, tumor grade III

Supplementary Table S1. Breast cancer plasma sources. The patients from Study A and Study B showed comparable age, metastasis, cancer type, tumor size and tumor grade. The healthy samples from both Study A and Study B were obtained from women with an average age of 47 years and a median of 49 years, which showed comparable mean ages with breast cancer samples in Study A and Study B (an average age of 52 years and a median of 52 years).
