# Supplementary material for: A method for identifying discriminative isoform-specific peptides for clinical proteomics application
Source: BMC Genomics. 2016 Aug 22;17(Suppl 7):522. doi: 10.1186/s12864-016-2907-8 (PMC5001247; doi:10.1186/s12864-016-2907-8)
Supplement: Additional file 2: Table S2. — 90 alternative splicing isoforms with statistically significant (q < 0.05) differences between normal breast and breast cancer samples in Study II (PDF 184 kb) [file 12864_2016_2907_MOESM2_ESM.pdf]

Supplementary Table S2 90 alternative splicing isoforms with statistically significant ($q < 0.05$) differences between normal breast and breast cancer samples in Study A

Sequence	Gene	Splicing	Qvalue	Status	Validated by Exon Array
VVFNVICLFWPIITK	ACSL3	E11	1.33E-02	health	Y
MNGYG(C)TPFR	ARHGAP26	E3_E22	1.66E-01	cancer	
DCKSSQGCWSGSQ^VIGIGAGQQSR	ATIC	E2_E14	2.09E-02	cancer	
YLMLLKELPGEEYPLPMEDVLELLKPL(S)YTVK	ATM	E19_E50	2.09E-02	cancer	
YLNWDAVF(R)LITYSLVIYQR	ATM	E3_E27	4.79E-02	health	
QGKYLNWDAVF(R)PSCIMDVSLR	ATM	E3_E30	2.09E-02	cancer	
QGKYLNWDAVF(S)WLHTWTW	ATM	E3_E59	5.61E-03	health	
CEILQLLKPMCE^SKR	ATM	E36_E45	8.95E-02	cancer	
QESF(R)YTVK	ATM	E42_E50	4.02E-02	cancer	
MGTKMMGGLGFHEVLNN	ATM	E51	2.09E-02	health	Y
MGTKMMGGLGFHEVLNN^K	ATM	E51_E54	1.09E-02	cancer	
IKFQIFGLFFQGYSVCE^SLMQCAGESITLFK	ATM	E6_E15	2.09E-02	cancer	
EVIIELFQLQIYIHHPKGAK	ATM	E7	2.09E-02	health	Y
ENLIELMADICHQ^IVPGHEGR	ATM	E8_E23	2.09E-02	cancer	
LGPAMPVSSPGFPGP(S)PAAAEGQLHGGAGGGGPQA APRLPVHRPASLHQAQEAER	BCR	E2_E10	2.09E-02	cancer	
NSLE(T)GPAGPAGQR	BCR	E7_E18	4.02E-02	cancer	
IGWPLFISNQSEVAEPEAASER	BCR	i18	2.09E-02	cancer	
GGWPCPIADLSFPGAGWCGR	BID	i3	1.09E-02	cancer	
FYSAHLFQNGSVLVGELYSYGTLL	BUB1	E21	1.09E-02	health	Y
HASLKPICQLK^GSELSGVISSACDK	BUB1	E5_E7	2.09E-02	cancer	
YCEGLFGSAILFIFILTLFTIFILALPFRL	BUB1	i17	2.53E-02	health	
ERDQ^SPAAPPGETAAPDAPR	CARD11	E8_E19	2.09E-02	cancer	
EVSVAAAAGPGFPAMADSSGQQ(G)DLSISADR	CARS	E1_E11	2.09E-02	cancer	
FLRAA(G)SYISFDILR	CARS	E1_E5	4.02E-02	cancer	
AFGYFEVTHDITKYSK	CAT	E3	4.02E-02	health	Y
KGGP(G)KPALAAAGAPALCTPGQADARPVLGP	CBFA2T3	E1_E6	4.02E-02	cancer	
PPALPMSFA(E)EAVNEVKR	CBFA2T3	i9_E10	1.08E-02	cancer	
(Q)VFLETLAPLAPLEQ	COL1A1	E1_E30	1.66E-01	cancer	
GNDGATGAAGPP^ESTGLTPTKAATWMPSK	COL1A1	E15_E49	4.02E-02	health	
GEPGPPGPAGAAGPA^GPAGPTGPVGPVGARGPA	COL1A1	E17_E44	2.09E-02	cancer	
GAN^GADGQPGAKGEPGDAGAK	COL1A1	E18_E36	1.66E-01	cancer	
GAPGIAGAPGFPGAR	COL1A1	E19	4.02E-02	health	Y
NCPGAEVPEGECCPVCPDGS(G)RER	COL1A1	E2_E31	1.36E-01	cancer	
SPTDQETTGVE^GDAGPKGADGSPGK	COL1A1	E3_E33	4.79E-02	cancer	
NDTLDPE(P)YVTGHIWACPPSEGDDYIFHCHPPDQK	CREBBP	E21_E27	2.09E-02	cancer	
ILGSPASGIQNTIGSVGTGQQNATSLSNPNPIDPSSMQR	CREBBP	E6	2.09E-02	health	Y
SVSSPTSSNTPTPTK	CYTSB	E2	4.02E-02	health	Y
WGLEANLLSQ^AMGPEEWLVQR	ELN	E4_E14	2.09E-02	cancer	

GLPDDELEEDQFGYWKR	ERCC6	E13	2.09E-02	health	Y
QIFKQFLTNR	ERCC6	E17	4.02E-02	health	Y
SGLVVQIT(G)TGSDVQTPKCHLK	ERCC6	E7_E18	2.09E-02	cancer	
CYLLYLTINHFIAVFCLLYLPFK	ERCC6	i3	5.61E-03	cancer	
MLK(W)PTACEVDGTR	FGFR1	E11_E15	2.09E-02	cancer	
LVSI(G)PRLVSNHSLHETSSVFVDSLTK	FLNA	E1_E44	2.09E-02	cancer	
VPVHDVTDASK	FLNA	E25	2.09E-02	health	Y
AYGP(G)DYEVSVKFNEEHIPDSPFVVPVASPSGDAR	FLNA	E4_E40	2.09E-02	cancer	
AP^DNEGCPVEALVKDNGNGTYSCSYVPR	FLNA	E9_E14	2.09E-02	cancer	
GDSNSSAGWK^H	FOXO4	E1_E3	1.22E-02	health	
GGQQQLQHGHPGGQPDQPPAGGSEPLQEHLEQQ	GNAS	E10	1.09E-02	health	Y
YLNQFENCCGLREGAILTLLSDI(G)K	IKBKB	E5_i5	4.02E-02	cancer	
EGIDSECGPFIKLSDPGIPITVLSR	JAK1	E16	4.02E-02	cancer	Y
GRVVSTEVDCR	JAK1	i14	6.38E-03	cancer	
NQLSNSTQ^PASINWQWNGTNGTNCK	KDM6A	E17_E27	2.09E-02	cancer	
CISV^HLEQLR	KDM6A	E5_E16	2.53E-02	cancer	
AEVQKLQALANEQ^ESSEK	KTN1	E16_E44	1.66E-01	cancer	
QNDQ^AQQSLELIQSKIVK	KTN1	E22_E43	4.79E-02	health	
GDLTIANLGTSEGRFMQ^YLVER	MET	E3_E10	8.95E-02	cancer	
QMCAIGGMPER	MET	E5	4.02E-02	health	Y
QSAHHLELMSLPIK	MET	i6	2.09E-02	cancer	
MPALSATPVPADETPETGLK	MLLT6	E10	4.02E-02	health	Y
TSRHSSGGGGGGAGGGGGSMGGGGSGFISGR	MLLT6	E7	2.09E-02	health	Y
TG(S)LDLLGDRSASSAGPQR	MPL	E8_E10	2.87E-03	cancer	
LLPNQNLPLDITLQSPTGAGPFPPIR	NCOA2	E13	1.66E-01	health	N
VNDPALRGGNLFPNQLPGMDMIK	NCOA2	E22	2.09E-02	health	Y
QESARNVLTNLDP(E)ILLTLLKPVASSADR	NCOA2	E3_E23	2.09E-02	cancer	
AAMKPGWEDLVRR	NCOA2	E9	8.95E-02	health	N
KLWTNCIILGEE(D)IK	NCOR1	E13_E28	2.09E-02	cancer	
S(G)IIDLTNMPPTILVPHPGGTSTPPMDRITYIPGTQITF PPRPYNSASMSP	NCOR1	E24_E34	1.09E-02	cancer	
TVLSGSIMQ(G)SVSSR	NCOR1	E29_E44	2.09E-02	health	
RSGSGNGLLQLPPTSTCGQ^QLLSQELR	NCOR1	E35_E43	2.09E-02	cancer	
TK(G)IIDLTNMPPTILVPHPGGTSTPPMDR	NCOR1	E9_E34	2.09E-02	cancer	
ECVLPTTMAQDTA(T)NSNPCEHAGKCVNTDGAFHCEC LK	NOTCH2	E2_E8	2.09E-02	cancer	
GADCTEDVDECAM(G)FTGPVCQIDIDDCSSTPCLNGA K	NOTCH2	E7_E10	4.02E-02	cancer	
WYPERVTQQ^SSEGTSPAQQQVALLDLQSALFCSQLEI QK	PDE4DIP	E2_E12	2.09E-02	cancer	
TVFFFFFFFFEMESCSVAQAGVQWR	PLK3	i3	3.22E-02	cancer	
ELVENSLDAGATNI(G)NLIK	PMS2	E2_E11	9.82E-03	cancer	
DWHLWCFVGKIMLVLCISEGYM	RPS6KB1	E12	1.09E-02	health	Y
LVENSTSSLSISV^PSQIFSWGFLGK	RPS6KB1	E5_E15	2.09E-02	cancer	
WHHEAWGAH^CPQR	SMO	E4_E12	5.19E-03	cancer	
RIMPEDIIINCS(N)LL	TOP1	E12_E15	2.09E-02	cancer	
IPPPHPS(G)VVK	TP53BP1	E16_E22	1.09E-02	cancer	

HLPNLQTHKENPVL^EFFR	TP53BP1	E2_E10	2.09E-02	cancer	
SNVSSPATPTASSSSSTTPTRK	TP53BP1	E23	2.09E-02	health	Y
SLSSYLSQTGQA(E)TPFHFTLPK	TP53BP1	E4_E13	2.09E-02	cancer	
SLSSYLSQTGQA(V)LEK	TP53BP1	E4_E20	4.02E-02	cancer	
CSFLPHSSFRPWQNAGSAAGK	TP53BP1	i23	2.09E-02	cancer	
MSNIPACFLGSAQSENVLTDIK	WRN	E16	4.02E-02	health	Y
GFIQEVGLPK	WRN	E18	9.82E-03	health	Y
DEIQ^SNLEKLYSYKPCDK	WRN	E20_E28	1.09E-02	cancer	
SVFEDDLPFLEFTGSIVYSYDASDCSFLSEDI(R)	WRN	E3_E35	2.09E-02	cancer	
